# Epidemiological investigation of the relationship between common lower genital tract infections and high-risk human papillomavirus infections among women in Beijing, China

**DOI:** 10.1371/journal.pone.0178033

**Published:** 2017-05-22

**Authors:** Dai Zhang, Ting Li, Lei Chen, Xiaosong Zhang, Gengli Zhao, Zhaohui Liu

**Affiliations:** 1 Department of Obstetrics and Gynecology, Peking University First Hospital, Beijing China; 2 Department of Obstetrics and Gynecology, Haidian Maternal & Child Health Hospital, Beijing, China; Rudjer Boskovic Institute, CROATIA

## Abstract

**Background:**

The incidence of lower genital tract infections in China has been increasing in recent years. The link between high-risk human papillomavirus (HR-HPV) and other sexually transmitted diseases (STDs) remains unclear.

**Methods:**

From March to October 2014, gynecological examinations and questionnaires were conducted on 1218 married women. Cervical secretions and vaginal swab specimens were tested for *Chlamydia trachomatis* (*CT*), *Neisseria gonorrhoeae* (*NG*), *Ureaplasma urealyticum* (*UU*), yeast, clue cells and HR-HPV.

**Results:**

Laboratory results were available for 1195 of 1218 married women. HR-HPV was detected in 7.0% of participants. Forty-seven percent of women had lower genital tract infections (LGTIs). *UU* was the most common infection (35.5%), followed by bacterial vaginosis (BV) (10.5%), yeast infection (3.7%), *CT* (2.2%), and *Trichomonas vaginalis* (1.7%). BV was associated with an increased risk of HR- HPV (*P* < 0.0001; odds ratio, 3.0 [95% CI, 1.7–5.4]). There was a strong correlation between abnormal cervical cytology and HR-HPV infection (*P* < 0.0001).

**Conclusions:**

The prevalence of LGTIs in Beijing is at a high level. It is clinically important to screen for the simultaneous presence of pathogens that cause co-infections with HR-HPV.

## Introduction

Lower genital tract infection (LGTI) is one of the most common causes of gynecological morbidities in China [1], jeopardizing health, economy, and quality of life [2,3]. Untreated LGTI such as *Chlamydia trachomatis* (*CT*) may ascend to the upper genital tract causing pelvic inflammatory disease (PID), which is commonly accompanied by serious reproductive complications including miscarriages, preterm births, ectopic pregnancy and tubal factor infertility [4,5].

Previous studies have reported the prevalence of 11.4–21% for the most common LGTIs in women of reproductive age [6]. However, with the development of new medical diagnostic techniques, more gynecologists in China have observed that the incidence of LGTIs has increased in recent years. Genital tract infections not only harm the health of the individual woman but also have an adverse effect on fertility, fetal growth, and development during pregnancy.

Bacterial vaginosis (BV) is the most prevalent LGTI in women of reproductive age throughout the world, and is a microecological imbalance of the genital tract in which the normal protective Lactobacilli-predominant microbiota are replaced by large numbers of anaerobic vaginal microorganisms [7]. Previous studies have demonstrated that BV was associated with an increased risk of preterm delivery, upper genital tract infection, sexually transmitted infections, HIV infection, and other conditions [8–10].

More than 30 bacterial, viral, fungal, and parasitic pathogens can be transmitted sexually, and among these human papillomavirus (HPV), *CT*, *Ureaplasma urealyticum* (*UU*), *Neisseria gonorrhoeae* (*NG*), and *Trichomonas vaginalis* (*TV*) are the most common causative agents of sexually transmitted diseases (STDs) [11]. *CT* is an obligate intracellular Gram-negative bacterium and comprises the most common bacterial STDs throughout the world [12]. Its prevalence is likely to be significantly underestimated since infection is often asymptomatic.

HPV is the most common viral sexually transmitted infection among women worldwide. The causal role of high-risk HPV (HR-HPV) in cervical carcinoma (CC) has been firmly established both biologically and epidemiologically [13]. Multiple non-HPV STDs may act as HPV co-factors in increasing the risk of developing cervical cancer [14]. However, there is no consensus on the association between HR-HPV and other STDs on CC progression [15].

The aim of this cross-sectional study was to evaluate the prevalence of HPV and other LGTIs in apparently healthy women in Beijing, China and identify factors associated with co-infections.

## Materials and methods

### Ethical approval of the study protocol

The study protocol was approved by the Ethics Committee of Peking University First Hospital, Beijing, China (2014[699]), and our study has been conducted in compliance with the ethical principles for medical research involving human subjects, as stated in the World Medical Association Declaration of Helsinki. Written informed consent was obtained for all eligible participants.

### Study subjects

A total of 1218 married women who underwent a routine health check for annual gynecologic examinations in Beijing were recruited to the study from March 2014 until October 2014. The inclusion criteria were as follows: the women had to be married, aged 20–70 years and residents of Beijing for at least 6 months. Exclusion criteria, according to our study protocol, were pregnancy, acute and chronic illness, lower genital tract malignancy, previous experience of cervical surgery or had been treated with previous pelvic radiation therapy.

### Gynecological examination and specimen collection

Gynecologists conducted regular gynecological examinations and collected vaginal and cervical specimens from all subjects. Each woman was allocated a unique identification number and the laboratory staff were blinded to clinical findings.

After the patients had given informed consent, cervical secretions were collected using aseptic endocervical cotton swabs. Cervical secretions were tested for HR-HPV, *CT*, *NG*, and *UU*. The vaginal swab specimens were rolled on to a glass slide for Gram staining. Laboratory tests for genital tract infection including *Trichomonas* in vaginal secretions were confirmed by wet mount. Gram staining of vaginal secretions for vaginal cleanliness [16] and detection of hyphae and spores of *Candida* and clue cells [17] were performed. Gram staining of cervical secretions for detection of *NG* and leukocytes was also conducted. The diagnosis of BV was based on both Gram staining and Amsel’s criteria [18].

### Interview survey

A standard risk factor assessment interview was conducted at the time of first visit for all 1218 enrolled subjects. Using a structured questionnaire, the trained physicians collected data on patients’ basic demographic status, reproductive health history, sexual behaviors and symptoms of genital tract infections, use of vaginal medications and use of contraceptive methods.

### *CT*/*NG*/UU testing

Cervical secretion specimens were tested for *CT*, *NG*, and *UU* using the *CT*/*NG*/*UU* nucleic acid test kit (HybriBio Ltd, Chaozhou, China). A PCR-fluorescent probe method (48 copies/box) was used to detect *CT*, *NG*, and *UU*.

### ThinPrep Cytological Test (TCT)

Cervical exfoliated cells were collected by cytobrush (Qiagen China Co., Ltd, Shanghai, China) from ectocervix and endocervix of the uterus of every woman by cervical scrapings. These were then fixed in TCT cytological solution for 15 min. ThinPrep 2000 (Hologic Inc., USA) and a SurePath liquid-based Pap test (BD, USA) were used for the TCT test. ThinPrep smears were screened by two independent cytopathologists. The smears were stained using standard Pap methodology and classified using the The Bethesda System (TBS; 2001) criteria [19,20] as follows: negative for intraepithelial lesion or malignancy (NILM); atypical squamous cells of unknown significance (ASCUS); atypical squamous cells-high grade (ASC-H); low-grade squamous intraepithelial neoplasia (LSIL); and high-grade squamous intraepithelial lesion (HSIL) or worse.

### HPV testing

HPV was tested using the *digene*^®^ HC2 High Risk HPV DNA Test^®^ (QIAGEN, Gaithersburg, MD) with the Rapid Capture System, which is based on signal amplification using RNA probes to target the entire HR-HPV genome [21]. All steps were performed according to the manufacturer’s protocols. Simply, cervical brush samples collected in preserve cytological solution underwent aprocess that included denaturation, hybridization, capture, and amplification of chemiluminescent signal detection. The HPV blot captures 21 HPV genotypes, including 6 low-risk types (HPV 6, 11, 42, 43, 44, and CP8304) and 15 HR types (HPV 16, 18, 31, 33, 35, 39, 45, 51, 52, 53, 56, 58, 59, 66, and 68) that are common in the Chinese population [22].

### Statistical analyses

All analyses were conducted with SPSS, version 13.0 (SPSS, Chicago, IL, USA). A *P*-value <0.05 was considered statistically significant. Pearson’s chi-square analysis was used to compare categorical variables between groups. Quantitative variables were compared between treatment groups using independent t-tests or non-parametric Mann-Whitney tests as appropriate. Associations between categorical variables were assessed using chi-square or Fisher’s exact tests (when appropriate). Data are reported as numbers (percentages) or odds ratios (ORs) with the corresponding 95% CIs.

## Results

### Prevalence of lower genital pathogens

Laboratory results were available for 1195 of 1218 married women (mean age 50.16 ± 6.82 years, age range 29–64 years). 23.4% (280/1195) of the participants underwent a Pap smear for the first time. Rates of the infections with the different organisms are shown in Table 1. Seven percent of otherwise healthy women were found to be HR-HPV-positive (84/1195), while notably, 47.1% of apparently health women had a LGTI (449/953). Of 953 otherwise healthy women, the prevalence of BV was 10.5% (100/953) and 3.7% for yeast infection (35/953). One point seven percent accounted for *Trichomonas vaginalis* (16/953), *CT* was identified in 2.2% (21/953), and *UU* was present in 35.5% (338/953) of women, while *NG* was not identified (Table 1).

**Table 1:** Prevalence of HR-HPV infection with respect to LGTIs.

Variable	No. ^a^	HR-HPV(-)	HR-HPV (+)	OR (95% CI)	*P* Value
**Total LGTIs**					
**Negative**^b^	504	471(93.5)	33(6.5)	1.512(0.942–2.425)	0.085
**Positive**^c^	449	406(90.4)	43(9.6)		
**Vulvovaginal candidiasis**					
**Negative**	918	844(91.9)	74(8.1)	0.691(0.163–2.938)	0.615
**Positive**	35	33(94.3)	2(5.7)		
**Bacterial vaginosis**					
**Negative**	854	796(93.2%)	58(6.8)	3.013(1.694–5.357)	<0.0001^d^
**Positive**	100	82(82.0)	18(18.0)		
***Trichomonas vaginalis***					
**Negative**	938	865(92.2)	73(7.8)	2.734(0.762–9.914)	0.108
**Positive**	16	13(81.2)	3(18.8)		
***Chlamydia trachomatis***					
**Negative**	932	860(92.3)	72(7.7)	2.810(0.921–8.574)	0.058
**Positive**	21	17(81.0)	4(19.0)		
***Ureaplasma urealyticum***					
**Negative**	615	568(92.4)	47(7.6)	1.134(0.700–1.839)	0.619
**Positive**	338	309(91.4)	29(8.6)		

^**a**^ one participant in 954 women with the laboratory results of vaginal swab specimens missed the data of *Candida*, *Chlamydia trachomatis* and *Ureaplasma urealyticum*.

^b^ Negative for all STD-causing micro-organisms.

^c^ Positive for any STD-causing micro-organisms including multiple infections.

^d^ *P* < 0.0001 (Chi-square tests).

Values are presented as number (% of row).

HR-HPV: high-risk human papillomavirus; LGTI: lower genital tract infections; OR = Odds Ratio for presence of pathogens in HR-HPV positive compared with HPV negative women; STD: sexually transmitted diseases.

### Co-infection with HPV and other microorganisms

Of the the afore-mentioned 953 otherwise healthy women, approximately 8.0% were HR-HPV positive (76/953). BV was more likely to be diagnosed in HR-HPV positive women [23.7%(18/76) vs. 6.6%(58/878) who were HPV negative], and a significant association between presence of HR-HPV and concurrent genital BV infection was observed (Table 1; *P* <0.0001, OR = 3.01). Statistical analyses did not reveal any association between presence of HR-HPV and yeasts, *Trichomonas vaginalis* or *UU* (*P* > 0.05). The prevalence of *CT* was 5.3%(4/76) among HR-HPV positive women, while 8.2% (72/877) among the HR-HPV negative women (*P* = 0.058). *Trichomonas vaginalis* is a common STD but it is not considered to be a co-factor of HPV in lesion progression to CC.

### Physical examination

Physical examination revealed that vulval abnormalities were observed in 14 women, accounting for 1.2% of total subjects. Vaginal wall abnormalities were observed in 43 (3.6%) subjects, and cervical abnormalities were found in 450 (37.7%) subjects (Table 2). Physical examination of uterus size revealed that hysterauxesis was observed for 109 (9.1%) subjects. Uterine tenderness was reported by 36 (4.2%) subjects. Abnormal uterine adnexa were identified in 36 (3.0%) subjects.

**Table 2:** Comparison of demographic, clinical correlates and reproductive history of HR-HPV or *CT* status.

	HR-HPV			*CT*		
	Negative	Positive	*P* Value	Negative	Positive	*P* Value
**Age/years**	50.18±6.83	49.85±6.78	0.661	50.18±6.90	46.71±4.41	0.022^a^
**Parturition frequency**	2.37±1.11	2.29±1.18	0.522	2.25±1.09	2.86±1.42	0.0138
**Spontaneous abortion/N**^b^**(% of row)**						
**Negative**	995(92.9%)	76(7.1%)	0.380	719(97.8%)	18(2.2%)	0.425
**Positive**	30(96.8%)	1(3.2%)		28(100.0%)	0 (0.0%)	
**Vulval abnormalities/N**^a^**(% of row)**						
**Negative**	1089(92.9%)	83(7.1%)	0.993	916(97.8%)	21(2.2%)	0.616
**Positive**	13(92.9%)	1(7.1%)		11(100.0%)	0(0.0%)	
**Cervical abnormalities/N**^a^ **(% of row)**						
**Negative**	660(93.2%)	48(6.8%)	0.521	549(98.0%)	11(2.0%)	0.435
**Positive**	415(92.2%)	35(7.8%)		354(97.3%)	10(2.7%)	
**Vaginal wall abnormalities/N**^a^ **(% of row)**						
**Negative**	1064(93.1%)	79(6.9%)	0.237	886(97.7%)	21(2.3%)	0.324
**Positive**	38(88.4%)	5(11.6%)		41(100.0%)	0(92.9%)	
**Abnormal vaginal discharge/N**^a^ **(% of row)**						
**Negative**	882(92.5%)	71(7.5%)	0.936	754(98.4%)	12(1.6%)	<0.0001^c^
**Positive**	133(92.4%)	11(7.6%)		93(93.0%)	7(7.0%)	
**Coital bleeding/N**^a^ **(% of row)**						
**Negative**	980(92.8%)	76(7.2%)	0.087	818(97.8%)	18(2.2%)	0.689
**Positive**	36(85.7%)	6(14.3%)		30(96.8%)	1(3.2%)	

^a^ *P* < 0.05.

^b^ Numbers did not always sum to the total due to missing data.

^c^ *P* < 0.001.

HR-HPV: high-risk human papillomavirus; *CT*: *Chlamydia trachomatis*.

### HR-HPV status and *CT* infections

HR-HPV status was associated with parturition frequency (*P* = 0.029, OR = 1.12, 95% CI: 1.01–1.24). Women with coital bleeding had a higher prevalence of HR-HPV infection than those without coital bleeding (14.3% vs. 7.2%), although the difference was not statistically significant (*P* = 0.122, Table 2).

**Fig 1:**
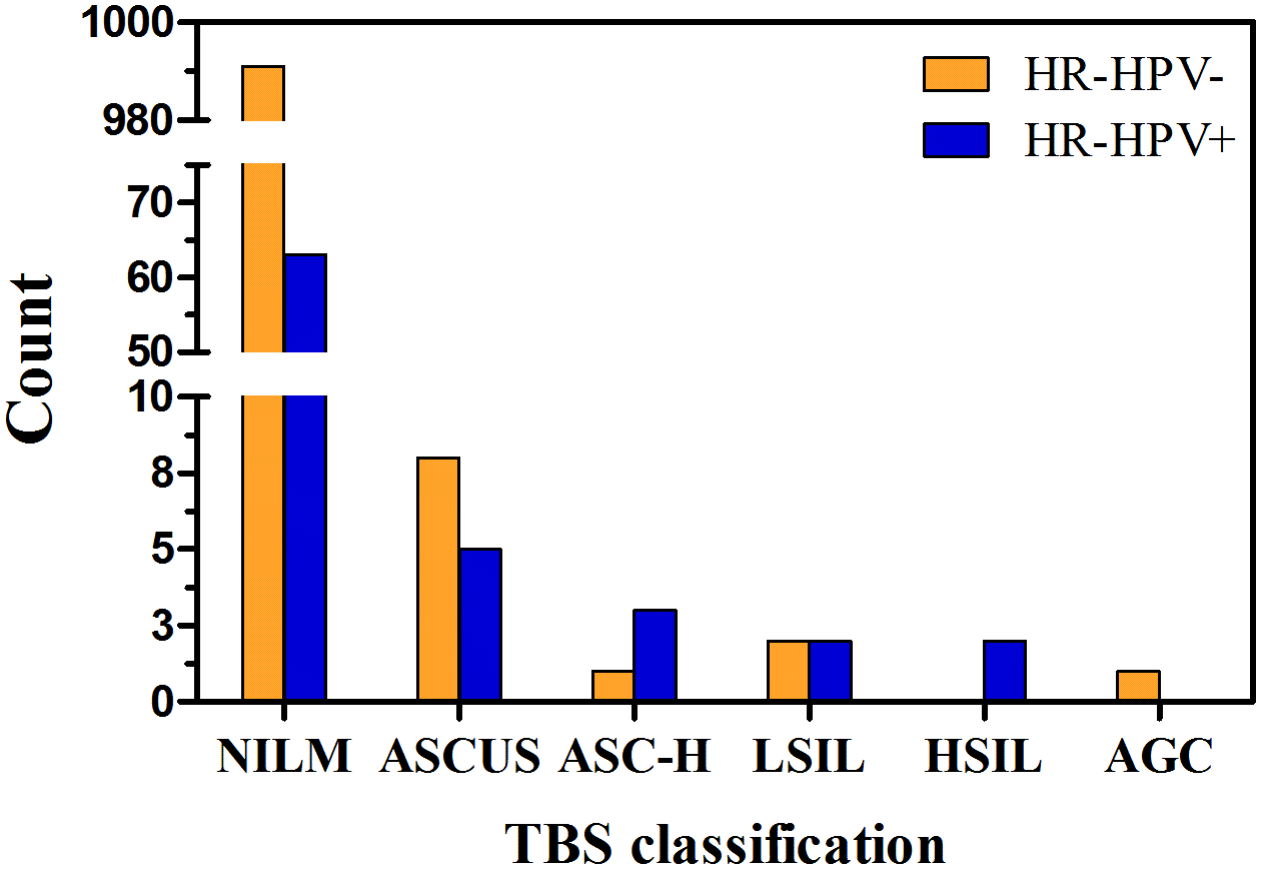
Number of HR-HPV positive and HR-HPV negative women by different abnormal cytological findings. HR-HPV: high-risk human papillomavirus; NILM: negative for intraepithelial lesion or malignancy; ASCUS: atypical squamous cells of unknown significance; ASC-H: atypical squamous cells-high grade; LSIL: low-grade squamous intraepithelial neoplasia; HSIL: high-grade squamous intraepithelial lesion; AGC: atypical glandular cells or worse.

Of 1195 specimens for HR-HPV testing, only 1078 participants have both valid cervical cytology and HPV infection data, while the data of other participants were invalid or missing. As expected, there was a strong correlation between abnormal cervical cytology and HPV infection (*P* < 0.0001, Table 3, Fig 1). Of the 1078 samples, 75 (7.0%) women presented with HR-HPV infection. In addition, the comparisons between cytological diagnosis and TBS classification demonstrated a strong association between HR-HPV DNA detection, which comprised the different classes of NILM, ASC-US, LSIL, ASC-H, HSIL, AGC and cancer (*P* < 0.0001, Table 3, Fig 1).

**Table 3:** Prevalence of HR-HPV infection or *CT* infections with respect to cervical cytology status.

TBS classification	HR-HPV/N(% of row)			*CT*/N(% of row)		
	Negative	Positive	*P* Value	Negative	Positive	*P* Value
**NILM**	991(94.0%)	63(6.0%)	<0.0001^a^	801(97.7%)	19(2.3%)	0.055
**ASCUS**	8(61.5%)	5(38.5%)		10(100.0%)	0(0.0%)	
**ASC-H**	1(25.0%)	3 (75.0%)		2(66.7%)	1(33.3%)	
**LSIL**	2(50.0%)	2(50.0%)		4(100.0%)	0(0.0%)	
**HSIL**	0(0.0%)	2(100.0%)		2(100.0%)	36(0.0%)	
**AGC**	1(100.0%)	0(0.0%)		1(100.0%)	0(0.0%)	
**Tumor**	0(0.0%)	100(100.0%)		1(100.0%)	0 (0.0%)	

^a^ *P* < 0.0001(Fisher's Exact Test);

HR-HPV: high-risk human papillomavirus; *CT*: *Chlamydia trachomatis*; NILM: negative for intraepithelial lesion or malignancy; ASCUS: atypical squamous cells of unknown significance; ASC-H: atypical squamous cells-high grade; LSIL: low-grade squamous intraepithelial neoplasia; HSIL: high-grade squamous intraepithelial lesion; AGC: atypical glandular cells or worse.

Furthermore, in our study, *CT*-infected women were significantly younger than *CT*-uninfected women (mean age ± SD: 46.71 ± 4.41 vs. 50.18 ± 6.90, *P* = 0.022), and parturition frequency was significantly higher among *CT*-infected women than *CT*-uninfected women (mean frequency ± SD: 2.86 ± 1.42 vs. 2.25 ± 1.09, *P* = 0.013). Moreover, there was strong evidence of an association between *CT* and abnormal vaginal discharge (*P* < 0.0001, OR = 0.211, 95% CI: 0.08–0.55). There was no significant difference in coital bleeding, parturition frequency, cervical hemorrhage or cervical erosion for those who were *CT*-positive/negative (*P* > 0.05). Infection with *UU* was associated with being *CT*-positive (*P* = 0.040) but *CT*-positivity was only weakly associated with HPV infection (*P* = 0.058).

## Discussion

Our present population-based study confirmed that disruption of the vaginal microbial community does occur amongst the healthiest women who come forward for physical examination, and that they experience very high rates of LGTI (47.1% of healthy women). Of these, the prevalence of sexually transmitted *CT* and *Trichomonas vaginalis* accounted for 2.2% and 1.7% of the sample, respectively. Generally, *UU* is a common commensal of the female lower genital tract and it seems to be an important opportunistic pathogen when combined with other genital diseases such as cervicitis [23]. The aforementioned pathogens may be transmitted from their sexual partner, which represents a higher risk of acquiring STDs, in an unfavorable scenario of difficult access for treatment of infection.

More than 40 closely related but genetically distinct HPVs that infect the genital tract have been classified as HR or low-risk (LR) according to their oncogenic potential [24–26]. Cervical HR-HPV infection is relatively common in China (7.0% of healthy women). Infection with HPV is a necessary precipitant of almost all cases of cervical cancer. Most HPV infections are undetectable within 2 years [27]. However, when the immune system is not able to control HPV infections, or other causal factors (such as other co-infections or immunodeficiency) exist, HR-HPV infection can persist for 12–15 years resulting in chromosomal instability, which may ultimately result in cervical intraepithelial neoplasia (CIN) [28]. Screening of abnormalities using a combination of cytology (Pap test) and testing of HPV by detection of HPV DNA or RNA has been the successful mainstay of cervical cancer prevention [29,30]. Primary prevention of HPV infection through immunization will help to prevent cervical cancer.

According to a HPV prevalence survey performed by WHO/IARC (World Health Organization/International Agency for Research on Cancer) in three areas of China, females without cervical abnormalities had an HPV 16, 52, and 58 infection rate of 10% to 14%, which was second only to that of sub-Saharan Africa [31]. WHO/IARC analyzed and summarized the global age distribution of HPV in 2006. India and China had similar age-specific infection with the highest infection rate in women aged 35 to 44-years old and the HPV infection rate showed no significant decrease as age increased, especially for HPV 16. A 10-year prospective study by Kjaer and colleagues [32] showed that the natural clearance rate of HPV in those infected before the age of 30 was higher than in those infected after the age of 30. As a result, women older than 30 are more likely to develop a persistent HPV infection thereafter, and in turn are more prone to cervical abnormalities. This is especially true as national HPV immunization programmes are associated with reductions in both low- and high-grade CIN in women < 25 years of age [29].

*CT* infection of the female genital tract can be passed by the mother to her newborn during delivery, and ranks first among all bacterial STDs [33]. The reported incidence of female genital tract *CT* infection differs throughout countries. The *CT* prevalence ranges between 0.2% in Spain and 5.6% in Nigeria [34]. The prevalence of *CT* infection in healthy women in our study was 2.2%, which is comparable to previous studies [34]. In our study, younger age and more childbirths led to enhanced susceptibility to *CT* infection, which suggested that age and parturition were associated with *CT* infection [35]. These data also support the finding that young adults are high-risk groups that are being infected with *CT* [34]. Moreover, our study have shown that *Trichomonas vaginalis* is not considered to be a co-factor of HPV in lesion progression to CC, which was essentially in agreement with other study [6].

In this study, the lack of significant association between current *CT* and HR-HPV infection and potential indicators may be partly due to inaccuracies in the self-reported information and absence of some relevant information (e.g., history of spontaneous abortion and coital bleeding). However, there have been several studies regarding the role of *CT* in the development of CC in HPV-infected women. A new meta-analysis study confirmed that individuals who were co-infected with HPV and *CT* had a higher risk of cervical cancer [36]. On one hand, *CT* infection induces a shift from a protective cellular (T helper cell 1, Th1) immune response to the proinflammatory humoral (Th2) immune response, which may adversely influence the clearance of HPV lesions [15]. Chronic cervical inflammation influences HPV persistence via a raised production of free radicals and reduction of host cell-mediated immunity [23,37]. On the other hand, *CT* infection may increase susceptibility to HPV causing microabrasions or alterations of epithelial cells thus facilitating the entry of HPV [23]. Considering that *CT* infection is asymptomatic in 70 to 75% of infected women [34], *CT* screening must be improved so that *CT* can be treated promptly. This is especially the case for those who are HR-HPV positive, since it will not only protect against pelvic inflammatory disease and infertility, but may also prevent CC from developing.

Vaginal colonization with Ureaplasma spp. occurs in 40–80% of women [38]. *UU* is a frequent cause of urethritis, vaginitis, cervicitis, spontaneous abortion, and infertility [39]. In our study, the *UU* prevalence rate in healthy women in our study was 35.5%, which is broadly comparable with previous studies [38,39]. The positivity rate and the pathogenic load of *UU* infection may be related to HR-HPV status and the pathogenesis of CC [40]. We speculate that the presence of *UU* may play a role both in initiating cellular anomalies and in viral persistence. However, our study does not support the association that *UU* infection is positively correlated with HR-HPV status, and further studies with a larger sample size are needed to confirm this issue and the potential mechanisms associated with these pathogens.

Our study suggests that HR-HPV infection was significantly associated with BV, indicating that BV may serve as a cofactor in the development of CIN. BV is a condition characterized by a decrease in *Lactobacillus spp*. with a concomitant increase in the diversity and numbers of anaerobic bacteria, including *Gardnerella vaginalis*, *Prevotella*, and *Atopobium vaginae*, which can delay the clearance of HPV [41,42]. Furthermore, the only prospective study on BV and HPV infection, showed that both infections occur simultaneously and the presence of HPV may also have an influence on the vaginal flora [43]. The vaginal microenvironment may be considered a cofactor in the pathogenesis of CIN [23], which causes inflammatory processes and microabrasions on the cervical epithelium, exacerbating the infection scenario and promoting the persistence of HPV. The mechanism of how bacterial, viral and other microbial pathogens collaboratively promote precancerous cervical lesions and cervical cancer should be further investigated. Our results suggest and emphasize the value of the screening and treatment for LGTIs in HR-HPV positive patients in order to reduce the probable synergistic effects of co-infections and to prevent the development of HPV-related cervical dysplasia.

## Conclusion

The prevalence of co-infections in our study population was of a magnitude that warrants attention by public health services. Our results reinforce the hypothesis that some non-HPV STDs might play a role as cofactors in HPV-mediated cervical carcinogenesis. It may be important to screen for the simultaneous presence of different co-infections with HPV in married women, especially young women. Futher studies could reveal the mechanisms by which the vaginal microbiota, as a community or through the actions of specific bacteria, provides protection against HR-HPV infection.
